# FastBLAST: Homology Relationships for Millions of Proteins

**DOI:** 10.1371/journal.pone.0003589

**Published:** 2008-10-31

**Authors:** Morgan N. Price, Paramvir S. Dehal, Adam P. Arkin

**Affiliations:** 1 Physical Biosciences Divison, Lawrence Berkeley National Laboratory, Berkeley, California, United States of America; 2 Virtual Institute for Microbial Stress and Survival, Berkeley, California, United States of America; 3 Department of Bioengineering, University of California Berkeley, Berkeley, California, United States of America; Pasteur Institute, France

## Abstract

**Background:**

All-versus-all BLAST, which searches for homologous pairs of sequences in a database of proteins, is used to identify potential orthologs, to find new protein families, and to provide rapid access to these homology relationships. As DNA sequencing accelerates and data sets grow, all-versus-all BLAST has become computationally demanding.

**Methodology/Principal Findings:**

We present FastBLAST, a heuristic replacement for all-versus-all BLAST that relies on alignments of proteins to known families, obtained from tools such as PSI-BLAST and HMMer. FastBLAST avoids most of the work of all-versus-all BLAST by taking advantage of these alignments and by clustering similar sequences. FastBLAST runs in two stages: the first stage identifies additional families and aligns them, and the second stage quickly identifies the homologs of a query sequence, based on the alignments of the families, before generating pairwise alignments. On 6.53 million proteins from the non-redundant Genbank database (“NR”), FastBLAST identifies new families 25 times faster than all-versus-all BLAST. Once the first stage is completed, FastBLAST identifies homologs for the average query in less than 5 seconds (8.6 times faster than BLAST) and gives nearly identical results. For hits above 70 bits, FastBLAST identifies 98% of the top 3,250 hits per query.

**Conclusions/Significance:**

FastBLAST enables research groups that do not have supercomputers to analyze large protein sequence data sets. FastBLAST is open source software and is available at http://microbesonline.org/fastblast.

## Introduction

Protein BLAST (basic local alignment search tool [1]) is often used to identify homologs for every sequence in the database, or “all-versus-all” BLAST. The resulting pairwise homologies are used to annotate protein sequences, to identify potential orthologs, and to identify new protein families. Another advantage of running all-versus-all BLAST and storing the results is so that users do not need to wait when they wish to view the BLAST results for a protein of interest: BLASTing a single protein against Genbank can take several minutes.

Unfortunately, all-versus-all BLAST is becoming computationally intractable. Analyzing a single metagenomics data set of 28.6 million protein sequences with all-versus-all BLAST required over 1 million hours of CPU time [2]. A research group with a cluster of 100 CPUs would have to wait over a year for the result. Because finding all pairs of homologous sequences in a database of *N* sequences takes O(*N^2^*) time, this problem will be even more severe in the future.

The sheer size of the output from all-versus-all BLAST is also a problem, as this also grows with square of the size of the database. We estimate that all-versus-all BLAST on the non-redundant subset of Genbank (“NR”), which currently contains about 6.5 million proteins and 2.2 billion amino acids, would generate 37 billion pairwise homology relationships and 1.8 terabytes of tab-delimited output.

One way to reduce the computational time for BLAST is to cluster similar sequences together first, as with CD-HIT [3]. CD-HIT uses a greedy approach to cluster unaligned sequences, and it quickly tests if two sequences are similar by counting the number of shared *k*-mers before trying to align them. If CD-HIT compares two sequences and finds that they are similar, it keeps the longer one as an “exemplar” for the cluster, and it need not compare the shorter one to other sequences. Thus, CD-HIT takes O(*NM*) time, where N is the number of sequences and M is the number of resulting clusters.

CD-HIT is orders of magnitude faster than BLAST for identifying sequences that are 65–99% identical. (CD-HIT can cluster at lower identity thresholds as well, but not as quickly.) As of July 2008, clustering the 6.23 million known proteins at 50% (“uniref50”, ftp://ftp.ebi.ac.uk/pub/databases/uniprot/uniref/uniref50) yields 1.99 million clusters. We estimate that computing these clusters required over 10,000 CPU-hours (scaling by O(*NM*) from test runs or from the results of [4]). Even after clustering, running all-versus-all BLAST on uniref50 would take another ≈6,000 CPU-hours (data not shown).

Another alternative to all-versus-all BLAST is to compare the sequences to models of known families instead of to each other. Each family is typically described by a position-specific PSI-BLAST matrix [1], [5] or a hidden Markov model (HMM) [6]. PSI-BLAST profiles and HMMs are available for many protein families [7], [8]. Comparing sequences to known families scales much better than all-versus-all BLAST: it takes O(*NF*) time, where *F* is the number of models (currently about 53,000 between InterPro and COG combined).

Unfortunately, the standard tools for HMM search, such as HMMer 2.3 (http://hmmer.janelia.org), are about 50 times slower than BLAST or PSI-BLAST (data not shown). PSI-BLAST is much faster than HMMer because it uses an index of *k*-mers to find short matches, and it only considers alignments around regions that contain two such short matches. HMMer 3 (due in late 2008) will also use this type of heuristic and is expected to be about 200 times faster than HMMer 2, or even faster than PSI-BLAST (http://hmmer.janelia.org). In the meantime, we use FastHMM to quickly identify members of known families (http://microbesonline.org/fasthmm). FastHMM uses PSI-BLAST with sensitive settings to find candidate members of a family and then uses HMMer 2 to select true hits and to align those candidates to the HMM. FastHMM is about 30 times faster than HMMer 2.3 and the resulting hits cover 98% of the amino acids that the HMMer hits cover (Supplementary Table S1).

The key limitation of the known families is that they are not complete: some proteins belong to families that are not yet described by a PSI-BLAST profile or an HMM. There are also some families that are so diverse that they are difficult to model accurately, and some members of these families are likely to be missed by the models. In practice, about a third of sequenced proteins have BLAST homologs that are not described by the families (see below). Thus, to find all of the homology relationships, BLAST is still required.

## Results

### Our Approach

We have developed FastBLAST, a more scalable replacement for all-versus-all BLAST. FastBLAST starts with members of known families and with a multiple sequence alignment for each family. FastBLAST uses the known families and their alignments to avoid doing unnecessary work, and it uses fast clustering to further reduce the amount of work.

The known families allow us to avoid work because they already capture most of the homology relationships. Two genes that belong to the same family are homologous, and there is no need to run BLAST to discover this. Conversely, most pairs of genes are not homologous, so we assume that if two genes belong to different families, then there is no need to compare them. We will show that this assumption works well in practice. Although the HMMs are imperfect, if two homologous regions are misclassified into different families by one source of models, they will usually be classified as homologs by a model from another source or by one of the additional families that FastBLAST creates.

**Figure 1:**
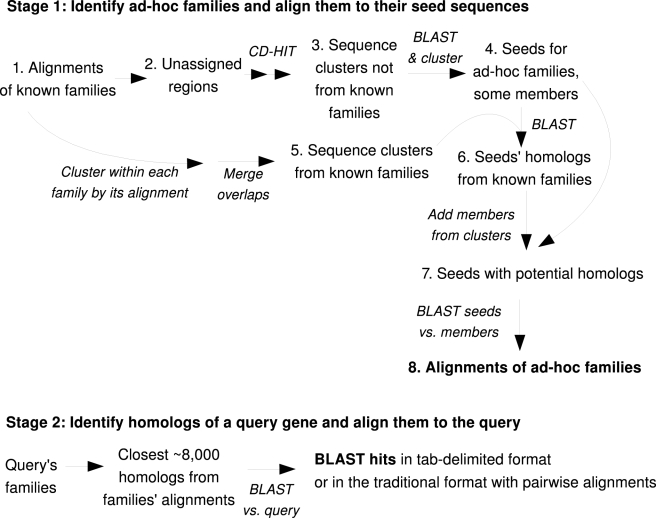
Overview of FastBLAST.

FastBLAST runs in two stages (Figure 1). First, it identifies “ad hoc” families that capture homology relationships that are missed by the known families. These ad hoc families are based on “seeds,” or unassigned regions that do not belong to any known family. The members of an ad hoc family are the homologs (from BLAST) of the seed. FastBLAST uses fast sequence clustering to identify these ad hoc families and their members quickly and to reduce the number of seeds. In the first stage, FastBLAST also creates multiple sequence alignments for the ad hoc families.

In the second stage, FastBLAST quickly finds the top homologs for a given gene by inspecting the alignments for the families that the query belongs to (both known families and ad hoc families). FastBLAST runs BLAST on just those top homologs instead of on the entire database. Thus, FastBLAST produces the same bit scores and pairwise alignments that NCBI BLAST does, and in the same output formats. However, if the families or their alignments are misleading, then FastBLAST may not identify all of the homologs that BLAST identifies.

Notice that we compute “top” homologs, rather than all homologs. Like BLAST, FastBLAST has a parameter that defines the number of homologs that are desired. However, unlike BLAST, FastBLAST runs more quickly if fewer homologs are desired. We recommend limiting the number of homologs identified to 1 per 2,000 sequences in the database: this should include all potential orthologs and all sequences with well-conserved functions. More distant homology relationships are better described using the domain families rather than with pairwise alignments. Our limit of 1/2,000 may not seem stringent, but some proteins are homologous to over 1/100 of all proteins (e.g., gi 16121781 has 107,873 homologs at 45 bits or above, which represents about 2% of Genbank NR).

Below, we describe FastBLAST in more detail, especially the key steps of identifying ad hoc families and selecting the top homologs of a gene. We then report the results of testing FastBLAST on NR.

#### Identifying Families

FastBLAST begins with known families and their alignments. FastBLAST can use families from any source that allows us to align the members to the family (e.g., HMMer or PSI-BLAST). In practice, we use raw HMM hits, as identified by FastHMM, to the families in Gene3D, PANTHER, Pfam, PIRSF, SMART, SUPERFAMILY, and TIGRFAMs [9]–[15]. We also use PSI-BLAST hits to COGs [8], [16]. For each family in the input, FastBLAST creates a multiple sequence alignment based on the profile-sequence alignments from FastHMM or PSI-BLAST. Positions that match the same profile position are aligned to each other, and positions that do not match the profile are removed. (In other words, insertions in the sequences, relative to the profile, are trimmed from the alignment.)

To identify the remaining families, FastBLAST finds homologs for unassigned regions that do not belong to any of the known families. The intuitive idea is to cluster the unassigned regions to obtain sequences that are potential seeds for new families, to use BLAST to find homologs for the seeds, and to create multiple sequence alignments for the resulting ad hoc families from the pairwise alignments to the seeds. If the HMMs were perfect models of the families, then we would only need to compare the seeds to other unassigned regions, but in practice, we need to compare the seeds to members of known families as well. FastBLAST uses clustering to reduce the number of sequences within the known families before it does this comparison.

The data flow of FastBLAST is shown in Figure 1. First, FastBLAST identifies unassigned regions that do not belong to any of the known families.

Next, to identify redundant sequences in the unassigned regions, FastBLAST uses CD-HIT [3] and BLAST. FastBLAST runs CD-HIT in two passes, first to cluster at 90% identity (with 5-mers) and then to cluster at 65% identity (with 4-mers). FastBLAST runs all-versus-all BLAST on the exemplars of the CD-HIT clusters and greedily clusters together sequences that are over 40% identical (see Methods for details). The sequences that remain after BLAST-based clustering are potential seeds for ad hoc families, and the BLAST hits (if any) of these seeds are members for these ad hoc families.

To identify redundant subsequences among the regions that belong to known families, FastBLAST uses a greedy approach to identify clusters of similar sequences. This method is similar to CD-HIT, but instead of counting *k*-mers, FastBLAST estimates sequence identity from the multiple sequence alignment. FastBLAST clusters together sequences whose aligned positions are over 33% identical (see Methods for details). FastBLAST also chooses an exemplar from each cluster. If overlapping regions of the same gene are exemplars for different families, then FastBLAST merges those regions. This is helpful because the databases of known families are highly redundant and many families overlap. For a given family, FastBLAST's alignment-based reduction is over an order of magnitude faster than CD-HIT and also gives a greater reduction (data not shown). Over all the families, FastBLAST should be even faster because FastBLAST only does comparisons within each family and need not compare members of different families to each other.

FastBLAST then uses BLAST to compare the non-redundant subset of unassigned regions (the seeds) to the merged non-redundant members of known families. Once this is complete, FastBLAST has homologs for the seeds from the seed's CD-HIT cluster, from the non-redundant unassigned regions, and from the merged non-redundant members of known families. Each unassigned region that has homologs other than itself (either from BLAST or from CD-HIT) is considered to define an ad-hoc family.

FastBLAST estimates the members of each ad hoc family by collecting the members of the seed's cluster, the seed's homologs, and the members of those homologs' clusters. FastBLAST then uses BLAST to compare each seed sequence to all of these potential members of the ad hoc family. This verifies that the genes are homologous to the seed and also gives pairwise alignments to the seed. Much like with the known families, FastBLAST uses these pairwise alignments to generate multiple sequence alignments. The final output of the first stage of FastBLAST comprises alignments for both known and ad-hoc families, the list of families for each gene, and indexes for rapid access to the list of families for a gene or to the alignment for a family (see Methods).

#### Selecting Top Homologs

To identify the top homologs of a gene, FastBLAST relies on the known families, the ad-hoc families, and the alignments. Naively, one could just select all potential homologs – genes that share a known family or an ad-hoc family with the query gene – and use BLAST to create pairwise alignments and select the top hits. This scheme works well for most genes, but for genes with very large numbers of homologs, it takes a long time to compute all the pairwise alignments.

To reduce the number of potential homologs considered, FastBLAST uses a heuristic based on the families' multiple sequence alignments. The assumption is that the top homologs of the gene should be top homologs according to the alignments. The alignments are imperfect and also do not cover all positions of the sequences, but we will show that this assumption works well in practice. FastBLAST computes a BLAST-like alignment score for the pairwise alignments between the query and its homologs that are implied by the families' alignments, and it selects the top 2.5*h* homologs, where *h* is the desired number of top homologs and 2.5 is an arbitrary safety factor.

Another complication is that some genes belong to many families with overlapping membership. In particular, the SUPERFAMILY and Gene3D databases contain many HMMs with overlapping specificity. The ad-hoc families are also likely to be redundant, as we only cluster the seeds to 40%. Thus, to save time, FastBLAST considers only the top few families for each region based on the bit scores of the hits (see Methods for details).

Once FastBLAST has selected the potential top homologs, it obtains their sequences from the BLAST database and runs BLAST to compute pairwise alignments and bit scores.

### Testing FastBLAST on NR

#### Performance of the First Stage on NR

To demonstrate that FastHMM and FastBLAST scale to large data sets, we ran them on the non-redundant Genbank database (“NR”). As of May 15, 2008, NR contained 6.53 million sequences of an average length of 342 amino acids, for a total of 2.23 billion amino acids. FastHMM identified members of known families in 8,552 CPU-hours; the time for PSI-BLAST to find hits to COGs was negligible, under 400 CPU-hours; and the first stage of FastBLAST took 5,509 CPU-hours. Together, these jobs took about 8 days to complete on a computer cluster with 160–192 CPUs available.

Most of the CPU time for the first stage of FastBLAST was in reducing the known families (≈1,000 CPU-hours), reducing the unassigned regions (≈1,500 CPU-hours), and comparing the potential seeds to the reduced regions from known families (≈2,300 CPU-hours). The third round of BLAST (aligning the seeds to the expected members of the ad hoc families) took less than 300 CPU-hours. The non-parallel steps took a total of 23 hours. The main bottlenecks were the two passes of CD-HIT clustering on the unassigned regions, which took a total of 15 hours. Optimizing the parallel version of CD-HIT might eliminate this bottleneck (we did not use the parallel version of CD-HIT because it did not reduce the elapsed time).

To compare the performance of FastBLAST to that of all-versus-all BLAST, we ran BLAST with 3% of NR as the query and NR as the database. This took 3,794 CPU-hours, so we estimate that all-versus-all BLAST on NR would take 3,794/0.03≈126,000 CPU-hours, or 23 times more work than the first stage of FastBLAST. This comparison does not include the time for FastHMM and PSI-BLAST to compare the database to the known families, but we think that this is justified because the family homologies are of great value in themselves.

We can estimate how much less work FastBLAST does, as compared to all-versus-all BLAST, from the size of the reduced forms of the NR database (Table 1). FastBLAST uses BLAST to compare the unassigned regions, clustered at 65%, to each other (14.1%⋅14.1% = 2.0% of the work of all-versus all BLAST). FastBLAST then compares the BLAST-clustered unassigned regions to the clustered/merged representatives of known families (11.3%⋅31.3% = 3.5% of the work of all-versus-all BLAST). The total work is 5.5%, so we would expect FastBLAST to be 18-fold faster (not considering the additional overhead of finding clusters, etc.). We believe that FastBLAST outperforms this theoretical speedup because there are relatively few significant alignments to find once the known families and the closely related sequences have been removed. FastBLAST produces only 17.8 million hits during the reduced BLAST runs, and 17.4 million total entries in the ad-hoc families, while all-versus-all BLAST would produce 37.1 billion hits. As the databases become larger and more redundant, the relative speed of FastBLAST should increase further, because the number of clusters should grow more slowly than the total database size.

**Table 1:** FastBLAST reduction of NR.

Step in Fig. 1	Subset	%Identity Threshold for Clustering	Sequences (millions)	Size (billions of amino acids)	Relative size	Alignments (millions)
–	All sequences	None	6.53	2.23	100.0%	–
1	Known families	None	–	1.72^d^	77.2%	214.7
5	Known families^a^	33%	2.28	0.70^e^	31.3%	–
2	Unassigned regions^b^	None	2.93	0.48	21.4%	–
–	Unassigned regions^c^	90%	2.20	0.37^e^	16.6%	–
3	Unassigned regions^c^	65%	1.86	0.32^e^	14.1%	–
4	Unassigned regions^c^	40%	1.49	0.25^e^	11.3%	–
8	Ad-hoc families	None	–	0.65^d^	29.2%	17.4
–	All families	None	–	2.13^d^	95.7%	232.1

a Sequence clusters from known families (clustered at 33% identity and merged).

b All “unassigned” regions of at least 30 amino acids that do not belong to any of the known families. FastBLAST ignores short linkers between two regions that belong to known families.

c Sequence clusters not from known families, clustered with CD-HIT or by analyzing BLAST hits.

d Total number of amino acids that belong to any of these families. Because of overlapping hits to families, this is far less than the total length of all the alignments.

e Total length of the exemplars of the clusters.

The first stage of FastBLAST was much faster than using CD-HIT to reduce the data set (we estimate that CD-HIT would take tens of thousands of CPU-hours) and about as fast as running all-versus-all BLAST on a reduced data set (we estimate that all-versus-all BLAST on uniref50 would take 6,000 CPU-hours). FastBLAST is faster than running CD-HIT on the entire data set because it does not compare sequences from different families to each other and because it uses a faster method to cluster sequences within a family.

#### Performance and Accuracy of the Second Stage on NR

**Figure 2:**
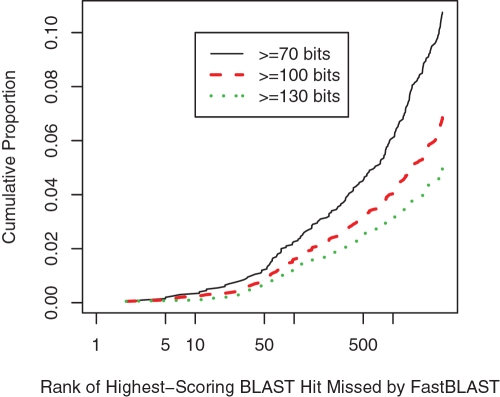
FastBLAST misses mostly low-ranking hits and/or weak hits. We show the cumulative proportion of queries that have a miss within the top *n* hits. Note the log-scale for the *x* axis. The highest proportion is 10.8% because FastBLAST identified all of the top 3,250 homologs at 70 bits or greater for the other 89.2% of queries. We also show results if only higher-scoring hits are considered.

To test the second stage of FastBLAST, we used both FastBLAST and BLAST to identify the top 3,250 hits for 2,000 randomly selected members of NR. (3,250 is 1/2,000 of the genes in NR.) BLAST took 40.8 seconds per query, while FastBLAST took 4.74 seconds per query, or 8.6 times faster. We believe that this is fast enough for interactive use (instead of pre-computing BLAST hits for every query). Among hits with scores of at least 70 bits, FastBLAST found 97.9% of the hits that BLAST found. As shown in Figure 2, FastBLAST correctly identified the top hit for every query (if the query had any homologs) and identified all 3,250 top homologs for all but 10.8% of the queries. For most of the remaining queries, the missed hits are weak or far down in the list. Thus, we doubt that the missed hits would be orthologs or would be useful for annotating the query's function.

If we did not use the ad-hoc families to select potential homologs (e.g., did not perform the first stage of FastBLAST) then the results would be dramatically worse: 33.4% of queries would have missing hits and 12.7% of queries would miss their top hit. This illustrates that although the known families capture the majority of the homology relationships, there are many additional relationships that are only captured by the ad-hoc families.

We examined in more detail the four queries for which FastBLAST missed a hit that was within the top 10 hits and over 100 bits. These queries and their worst missed hit are listed in Supplementary Text S1. One of the top hits would have been missed by other approaches to reduce the work of BLAST by clustering: a hit from A to B was missed because we clustered B with C, and B and C are 41% identical over the relevant region, and yet A does not hit C. For another top hit, the homologous regions identified by BLAST are repeats of VxSxxHGT. The two repeats have expanded independently, so we are not even sure if the sequences are truly homologous, even though the alignment score is 160 bits. The remaining two cases were relatively weak hits (108 and 103 bits) that were not captured by the alignments to known families. Improvements to the HMM search tools could eliminate these misses.

## Discussion

### Future Work

#### FastBLAST Features

The major feature that FastBLAST lacks is the ability to identify homologs for a query that is not in the database. If you have a large number of new sequences, such as newly sequenced genome(s), then FastHMM and FastBLAST can efficiently add the new sequences to an existing database (see Methods). However, FastBLAST does not have a way to quickly find homologs for a single new query. In principle, this could be done by comparing the query to the known and to the ad-hoc families, and then using the resulting alignments to select potential homologs. A query can be compared to ad-hoc families by running BLAST against the seeds of the ad-hoc families, which is much faster than running BLAST against the entire database (data not shown). However, we do not know of an efficient way to compare a single sequence to the known families. We experimented with using reverse PSI-BLAST to do so, followed by confirming hits with HMMer, but this was not much faster than running BLAST against the database (data not shown). With the expected performance gains from HMMer 3, this approach may become more attractive.

For gene-finding and for annotating metagenomics data, it is desirable to use nucleotide sequences as queries, as in blastx [17]. Significant speed-ups over blastx might be achieved by comparing the six-frame translation of the query to the known families (e.g., with the nucleotide mode of reverse PSI-BLAST) and then masking out regions that have strong hits to a known family (e.g., analyzing those regions in only one frame).

#### Performance Improvements

It may be possible to speed up the second stage of FastBLAST significantly. Identifying potential homologs by inspecting the alignments of the families takes an average of only 0.8 of the 4.7 seconds per query. Most of the time is spent retrieving the sequences of the candidate homologs and aligning them with BLAST. Retrieval time could be greatly reduced by using an in-memory database instead of using fastacmd to retrieve them from a BLAST database. The time to align the homologs to the query might be reduced by using the alignments implied by the families as a starting point to search for local alignments, instead of using BLAST to realign the homologs to the query.

In the first stage of FastBLAST, it should be possible to further reduce the members of known families. On NR, the clusters from known families are somewhat redundant because cluster exemplars are chosen separately for each family, and the families themselves are redundant. Because similar exemplars will be members of each others' clusters, it may be possible to identify these redundancies efficiently. However, comparing the potential seeds to the known families was only 45% of the CPU time for the first stage, so further reductions of the known families would not yield a dramatic performance improvement.

A more promising approach to speeding up the first stage might be to improve the models of known families. Many of the seeds are probably unrecognized members of known families: about a third of the regions that are members of ad hoc families are covered by known families as well. Devising PSI-BLAST profiles for the larger of the ad hoc families might also improve coverage: about half of the hits to ad hoc families are from families with over 100 members, and these larger ad hoc families amount to only ≈27,000 seeds. Identifying homologs for these seeds would take around 300 CPU-hours and would reduce the uncovered regions by around 25%, which should give a corresponding reduction in effort during the first stage (≈1,400 CPU-hours of savings). FastBLAST would also need to reduce these additional families, but the CPU time would be negligible, as these additional families have an average of only 325 members.

#### Orthologs

Besides functional annotation, a major use of BLAST hits is to identify potential orthologs. Orthologs are usually identified from bidirectional best BLAST hits, which requires doing all-versus-all BLAST. Although the resulting orthologs are often spurious [18], [19], more careful clustering-based analyses of the BLAST hits yield better results [20], [21]. Nevertheless, all-versus-all BLAST will not scale to thousands of genomes, because both the CPU time and the disk space required grow quadratically with the number of genomes.

Instead, we recommend building a phylogenetic tree for every family (including the ad-hoc families), and then using the trees to identify potential orthologs and to propagate annotations (e.g., [22]). Although some families now have over 100,000 members, trees of this size can be constructed in a few hours of CPU time (http://www.microbesonline.org/fasttree). A potential challenge is to reconcile the results from multiple families: the average gene in NR belongs to 33 known families and 2.7 ad-hoc families.

### Conclusions

We have shown that FastBLAST scales to databases with millions of proteins. The first stage of FastBLAST identifies additional families over 20 times faster than all-versus-all BLAST. These additional families should be useful for improving the databases of sequence families, either to suggest new families to add or, if the ad-hoc family overlaps with a known family, to improve the model of the family.

The second stage of FastBLAST identifies homologs for the average protein in NR in an average of five seconds, which supports rapid browsing of the sequence databases and eliminates the need to pre-compute BLAST hits. Although FastBLAST misses some of the homologies that are found by traditional BLAST, these tend to be weak or low-ranking hits. In many applications, these misses will not matter. Furthermore, FastBLAST finds most of the homologies that are not represented in the protein family databases. As the family databases improve, the sensitivity of FastBLAST and its speed relative to that of BLAST should also improve.

In combination with performance improvements to HMM search (e.g., FastHMM or HMMer 3) and with scalable methods for constructing phylogenetic trees (e.g., FastTree), FastBLAST enables a wide variety of analyses on large protein sequence databases, such as identifying orthologs, studying evolutionary histories, and predicting protein functions. All of these tools run in less than O(*N^2^*) time, and so it should continue to be feasible to run these analyses on a modest-sized computer cluster, despite the rapid growth of the sequence databases.

Source code for FastHMM and FastBLAST and results for the May 15 2008 release of NR are available at http://microbesonline.org/fastblast. FastBLAST is also being incorporated into the MicrobesOnline web site.

## Materials and Methods

### FastBLAST implementation

FastBLAST is mostly implemented in Perl. Two performance-critical steps are implemented in C: clustering the sequences in a family's alignment and identifying top hits to a gene given a family's alignment. FastBLAST requires about as much memory as the size of the database (about 2 GB for NR). During the first stage of FastBLAST, we use UNIX sort to avoid using a database or loading large data sets into memory. For the second stage of FastBLAST, which requires quick access to the alignment for a family and the families for a gene, FastBLAST uses BerkeleyDB, a light-weight open-source database, to store the indexes (http://www.oracle.com/technology/products/berkeley-db).

The first stage of FastBLAST is highly parallel and uses SunGridEngine's qmake, a variant of GNU make, to coordinate the execution of the jobs. If your compute cluster does not support parallel make, you can still use GNU make to generate lists of independent commands at each step.

### FastBLAST reduction

Here we give technical details of the reduction steps. When identifying unassigned regions, FastBLAST ignores unassigned stretches of ≤30 amino acids, as these short stretches are of limited use for finding homologs.

When using BLAST to cluster the unassigned regions, FastBLAST examines the results of all-versus-all BLAST (in arbitrary order). If the subject is over 40% identical to the query and the alignment covers at least 80% of the subject, then the subject is clustered with the query, and any homology relations involving the subject will be ignored. To ensure that a sequence that has homologs is not removed, FastBLAST keeps track of which sequences have been removed due to which exemplars. For example, if B is clustered with A, and then A is clustered with C, FastBLAST checks that B is a homolog of C before ignoring A and its homologs.

When clustering sequences within a family's alignment, FastBLAST analyzes the sequences with the fewest gaps first, and always uses the longest (fewest-gaps) sequence as the exemplar. (This is analogous to CD-HIT analyzing the longest sequences first.) When FastBLAST compares a potential cluster member to an exemplar, it ignores positions that are gaps in both sequences or just in the potential member (these can be thought of as truncations). Positions that are gaps in the exemplar but not in the potential cluster member are counted as differences. The member is assigned to the cluster if the two sequences are over 33% identical. To eliminate problems due to domain shuffling, FastBLAST also requires that both the N- and C-terminal 40 amino acids of the aligned regions be at least 30% identical.

### Selecting top homologs

To select candidates for the top homologs for a query, FastBLAST examines alignments for the query's families. However, to save time, FastBLAST does not examine every family's alignment. FastBLAST uses all hits from PFam, TIGRFAMs, SMART, and PIRSF, and the best hit from COG. FastBLAST adds other hits (best bit score first) until it reaches two hits to known families per region. Similarly, FastBLAST uses up to two hits to ad-hoc families per region. FastBLAST considers two hits to be potentially redundant if they overlap by more than 50%.

### Adding sequences to a FastBLAST database

Suppose that you already have a large FastBLAST database and you wish to add newly sequenced genomes to it. The first step is to run FastHMM and FastBLAST on the new sequences. Then, you can use the merge feature of FastBLAST to merge the two FastBLAST databases.

During a merge, FastBLAST uses BLAST to compare the potential seeds from the first database (the non-redundant subset of unassigned regions) to the non-redundant subset of the second database (including both unassigned and assigned regions), and vice versa. This gives potential new members of ad-hoc families, including hits for potential seeds that did not have homologs within their own database. Then, FastBLAST selects additional potential members for these ad-hoc families, based on the clusters. FastBLAST also removes redundant families: if seed sequence A for an ad-hoc domain from the second database is at least 45% identical to seed sequence B for an ad-hoc domain from the first database, and if the alignment covers at least 85% of A, then ad-hoc domain A is removed and its members are considered as candidates for ad-hoc domain B. Given the candidates, FastBLAST uses BLAST to confirm the membership of the sequences in the ad-hoc families and to align them to the seeds. Finally, it combines the alignments from the original FastBLAST databases with the new alignments to produce a new FastBLAST database. Because FastBLAST's merge operates on reduced sets of sequences as ordinary FastBLAST does, it should give a similar speed-up over BLAST and similar accuracy.

To test FastBLAST merge, we ran it on a randomly selected 25% and 2.5% of NR. The merge took 122 CPU-hours for the BLAST steps, followed by an hour for combining the databases. In contrast, we estimate that BLASTing one database against the other would have required about 790 CPU-hours. To verify that the resulting database was correct, we ran FastBLAST on the combined set of sequences as well, and we selected top homologs for 2,000 genes using either FastBLAST database. The two databases produced very similar results: for example, if we considered only the top 100 hits and only hits at 100 bits or higher, then for 99.5% of the queries, FastBLAST with the merged database found all of the hits that were found by FastBLAST with the combined database.

### Identifying known families with FastHMM

To force PSI-BLAST to find very weak homologs, FastHMM uses blastpgp with the options “-z 1e8 -Y 1e8 -e 10 -v 1000000 -b 1000000.” The -Y option reduces the search space size and hence PSI-BLAST will try to extend pairs of very weak hits. After identifying candidate members of families with PSI-BLAST, FastHMM uses fastacmd to extract the full gene sequences and HMMer's hmmsearch to validate the hits. FastHMM uses the -Z option to scale the E-values up by the number of families within each database. FastHMM's thresholds are similar to those of InterProScan: for Pfam, the gathering cutoff defined by the curators; for TIGRFAMs, the trusted cutoff; for SMART, per-protein ; for GENE3D and PANTHER, *E*<0.001; for SUPERFAMILY and PIRSF, *E*<0.02. For some families, blastpgp has poor sensitivity, so FastHMM simply runs hmmsearch against all sequences.

To find regions that are homologous to COGs, we used reverse PSI-BLAST with an E-value cutoff of.

### Computers

We ran FastHMM and the first stage of FastBLAST on a cluster with 48 nodes and 192 CPUs. Each node has two dual-core 2.2 GHz Opteron CPUs and 8–16 GB of RAM. We also ran HMMer on 3% of NR and BLASTed 3% of NR versus NR on this cluster.

We ran the second stage of FastBLAST, and the corresponding BLAST runs of those queries against NR, on a computer with a 2.4 GHz Intel Q6600 quad-core CPU and 8 GB of RAM. Both runs used a single thread of execution. We did not use the cluster because the nodes have only 60 GB of local disk space available, and the FastBLAST database for NR requires 79 GB (mostly for the alignments of the families). Because many of the family alignments are quite large (tens of megabytes), running these queries in parallel on the cluster would have overwhelmed the cluster's file server.

### Versions of protein families and of software

We used NCBI BLAST version 2.2.17, HMMer 2.3.2, and CD-HIT 2007. We used COG from Oct. 2006, Pfam version 20.0, TIGRFAM version 6.0, SMART 06_07_2006, Panther version 6.0, PIRSF from Dec. 7 2006, SUPERFAMILY version 1.69, and Gene3D from Dec. 11 2006.

### Settings for BLAST

We ran BLAST with composition-based statistics (the default for version 2.2.17), an effective database size of 10^8^, an E-value cutoff of 0.001 (corresponding to a minimum alignment score of 42 bits), and an unlimited number of hits. We masked low-complexity sequences for look-up but not for alignment (-F “m S”).

## Supporting Information

Table S1 Comparison of FastHMM to HMMer 2.3 on 3% of NR
(0.02 MB PDF)

Text S1 Homology Relations Missed by FastBLAST
(0.03 MB PDF)

## References

[1] Altschul SF, Madden TL, Schaffer AA, Zhang J, Zhang Z (1997). Gapped BLAST and PSI-BLAST: a new generation of protein database search programs.. Nucleic Acids Res.

[2] Yooseph S, Sutton G, Rusch DB, Halpern AL, Williamson SJ (2007). The sorcerer II global ocean sampling expedition: Expanding the universe of protein families.. PLoS Biol.

[3] Li W, Jaroszweski L, Godzik A (2002). Tolerating some redundancy significantly speeds up clustering of large protein databases.. Bioinformatics.

[4] Suzek BE, Huang H, McGarvey P, Mazumder R, Wu CH (2007). UniRef: comprehensive and non-redundant UniProt reference clusters.. Bioinformatics.

[5] Schaffer AA, Aravind L, Madden TL, Shavirin S, Spouge JL (2001). Improving the accuracy of PSI-BLAST protein database searches with composition-based statistics and other refinements.. Nucleic Acids Res.

[6] Durbin R, Eddy S, Krogh A, Mitchison G (1998). Biological sequence analysis: probabilistic models of proteins and nucleic acids.

[7] Mulder NJ, Apweiler R, Attwood TK, Bairoch A, Bateman A (2007). New developments in the InterPro database.. Nucleic Acids Res.

[8] Marchler-Bauer A, Anderson JB, DeWeese-Scott C, Fedorova ND, Geer LY (2003). CDD: a curated Entrez database of conserved domain alignments.. Nucleic Acids Res.

[9] Pearl F, Todd A, Sillitoe I, Dibley M, Redfern O (2005). The CATH domain structure database and related resources Gene3D and DHS provide comprehensive domain family information for genome analysis.. Nucleic Acids Res.

[10] Mi H, Lazareva-Ulitsky B, Loo R, Kejariwal A, Vandergriff J (2005). The PANTHER database of protein families, subfamilies, functions and pathways.. Nucleic Acids Res.

[11] Finn RD, Mistry J, Schuster-Böckler B, Griffiths-Jones S, Hollich V (2006). Pfam: clans, web tools and services.. Nucleic Acids Res.

[12] Wu CH, Nikolskaya A, Huang H, Yeh LS, Natale DA (2004). PIRSF: family classification system at the Protein Information Resource.. Nucleic Acids Res.

[13] Letunic I, Copley RR, Pils B, Pinkert S, Schultz J (2006). SMART 5: domains in the context of genomes and networks.. Nucleic Acids Res.

[14] Wilson D, Madera M, Vogel C, Chothia C, Gough J (2007). The SUPERFAMILY database in 2007: families and functions.. Nucleic Acids Res.

[15] Selengut JD, Haft DH, Davidsen T, Ganapathy A, Gwinn-Giglio M (2007). TIGRFAMs and Genome Properties: tools for the assignment of molecular function and biological process in prokaryotic genomes.. Nucleic Acids Res.

[16] Tatusov RL, Natale DA, Garkavtsev IV, Tatusova TA, Shankavaram UT (2001). The COG database: new developments in phylogenetic classification of proteins from complete genomes.. Nucleic Acids Res.

[17] Gish W, States DJ (1993). Identification of protein coding regions by database similarity search.. Nature Genet.

[18] Koski LB, Golding GB (2001). The closest BLAST hit is often not the nearest neighbor.. J Mol Evol.

[19] Price MN, Dehal PS, Arkin AP (2007). Orthologous transcription factors in bacteria have different functions and regulate different genes.. PLoS Comput Biol.

[20] Remm M, Storm CEV, Sonnhammer ELL (2001). Automatic clustering of orthologs and in-paralogs from pairwise species comparisons.. J Mol Biol.

[21] Dehal PS, Boore JL (2006). A phylogenomic gene cluster resource: the Phylogenetically Inferred Groups (PhIGs) database.. BMC Bioinformatics.

[22] Zmasek CM, Eddy SR (2002). RIO: Analyzing proteomes by automated phylogenomics using resampled inference of orthologs.. BMC Bioinformatics.

