# Supplementary material for: FastBLAST: Homology Relationships for Millions of Proteins
Source: PLoS One. 2008 Oct 31;3(10):e3589. doi: 10.1371/journal.pone.0003589 (PMC2571987; doi:10.1371/journal.pone.0003589)
Supplement: Table S1 — Comparison of FastHMM to HMMer 2.3 on 3% of NR (0.02 MB PDF) [file pone.0003589.s001.pdf]

Supplementary Table S1: Comparison of FastHMM to HMMer 2.3 on 3% of NR.

For each database of HMMs, we report the proportion of amino acids that are covered by the raw hits from `hmmsearch` 2.3.2 or from FastHMM. The coverage ratio is the fraction of amino acids covered by HMMer that are covered by FastHMM. We also show the proportion of raw hits or of family assignments that FastHMM finds (the sensitivity) and the total CPU time for both methods in hours. Family assignments are similar to InterProScan assignments. Roughly speaking, they keep for each region in the sequence the best hit within that database of HMMs.

	**Coverage**			**Sensitivity**		CPU Time (hr)	
**Database**	**HMMer**	**FastHMM**	**Ratio**	**Raw**	**Assign.**	**HMMer**	**FastHMM**
Gene3D	37.34%	37.24%	99.73%	98.89%	99.91%	721	12
PANTHER	42.67%	41.12%	96.35%	97.05%	94.98%	1,947	86
Pfam	50.42%	50.20%	99.56%	98.02%	99.41%	2,447	69
PIRSF	17.80%	15.30%	85.95%	96.21%	99.99%	365	16
SMART	9.92%	9.91%	99.90%	99.91%	99.92%	58	5
SUPERFAMILY	54.26%	53.14%	97.93%	94.49%	97.77%	1,176	20
TIGRFAMs	15.30%	15.30%	99.97%	99.88%	99.89%	729	22
Total	75.89%	74.50%	98.17%	95.66%	98.42%	7,442	230
