# Supplementary material for: FastBLAST: Homology Relationships for Millions of Proteins
Source: PLoS One. 2008 Oct 31;3(10):e3589. doi: 10.1371/journal.pone.0003589 (PMC2571987; doi:10.1371/journal.pone.0003589)
Supplement: Text S1 — Homology Relations Missed by FastBLAST (0.03 MB PDF) [file pone.0003589.s002.pdf]

Supplementary Text S1: Homology Relations Missed by FastBLAST.

Among our 2,000 test queries, there were four queries for which FastBLAST missed a hit that was within the top 10 hits and over 100 bits. For these four worst misses, we show the Genbank ids (gi #s) for the query and the missed subject, the rank (e.g. 124519843 is the 7th best hit of 71897758 other than itself), the alignment from BLAST, and a brief comment. Apart from the highly repetitive hit, which we suspect is spurious, the earliest rank is #5 and the highest score is 108 bits.

Query: 71897758, Subject: 124519843, Rank: #7

Clustering fails: subject is clustered with 149199875 (41% identical over a.a. 22-394), but 149199875 is not a BLAST hit of the query.

```
 Score =  105 bits (263), Expect = 3e-22,   Method: Composition-based stats.
 Identities = 61/175 (34%), Positives = 92/175 (52%), Gaps = 4/175 (2%)

Query: 5   QWDSASVADCLVTVPTAGTTKVQTRNYKAARRFPVIDQGRNQIAGWTDDEGAVINAP-FP 63
           +W+  S +D  +        +++T  Y  + ++PV+DQG+ ++  +++DE  V   P
Sbjct: 220 EWEKVSFSDIFIKTKVK-KHQIKTNEYLESGKYPVVDQGQKKVTAYSNDEEKVFEVPETG 278

Query: 64  LIVFGDHTRAFKFVKRSFARGADGIQLLRPKSGIDPLFFYACRAID-LPARGYNRHFTIL 122
           +IVFGDHTR  KF+   F  GADG Q+L  K   D  F+Y    I  +P  GYNRHF  L
Sbjct: 279 VIVFGDHTREIKFIDFDFIIGADGTQVLMTKDDYDVRFYYYHLLIQKIPNTGYNRHFKFL 338

Query: 123 KEKELTFPRDIDEQAAIAEVLRRTEHTLGKQAQILRALHDLKRATMRQLFTCGLR 177
           KE     P  + EQ AI+ +L   +  L      L AL++ K+  M+ L T  +R
Sbjct: 339 KEMIFNKP-SLKEQKAISNLLSTIDKELDLLNAELSALNEQKKGLMQLLLTGKVR 392
```

Query: 145475943, Subject: 71407532, Rank: #2

The sequences are highly repetitive. Because the query's repeat has the spacer EGE and the subject's repeat has the spacer STP, the repeats must have expanded independently. The similarity within each repeat is a maximum of only 5 amino acids in a span of 8, so the sequences might not even be homologous. We show only the first of 8 alignments from BLAST for this pair of sequences.

```
 Score =  157 bits (397), Expect = 4e-37,   Method: Composition-based stats.
 Identities = 123/421 (29%), Positives = 126/421 (29%)

Query: 440  SEEHGTTEGEGQSEDHGTQEGEGKSDEHGTTEGEGQSEDHGTQEGEGKSEDHGTTEGEGQ 499
            S  HG       S  HGT      S  HGT      S  H T      S  H T
Sbjct: 690  SSAHGAPSTPADSSAHGTPSTPVDSSAHGTPSTPADSSAHSTPSTPADSSAHSTPSTPAD 749

Query: 500  SEDHGSQEGEVKSDEHGTTEGEGQSEDHGTTEGEGKSEDHGTTEGEGQSQDHGTTEGEGQ 559
            S  H +    V S  HGT      S  H T      S  HGT      S  H T
Sbjct: 750  SSAHSTPSTPVDSSAHGTPSTPADSSAHSTPSTPADSSAHGTPSTPVDSSAHSTPSTPVD 809

Query: 560  SEDHGTQEGEVKSDEHGTTEGEGQSEDHGTQEGEGKSEDHGTTEGEGQSEDHGTTEGQGQ 619
            S  HGT    V S  H T      S  HGT      S  H T      S  H T
Sbjct: 810  SSAHGTPSTPVDSSAHSTPSTPVDSSAHGTPSTPVDSSAHSTPSTPADSSAHSTPSTPAD 869
```

```
Query: 620  SEDHGTQEGEVKSDEHGTTEGEGQSEDHGTQEGEVKSEDHGTTEGEGQSEDHGTQEGEVK 679
            S  HGT    V S  H T      S  H T    V S  H T      S  HGT    V
Sbjct: 870  SSAHGTPSTPVDSSAHSTPSTPADSSAHSTPSTPVDSSAHSTPSTPADSSAHGTPSTPVD 929

Query: 680  SDEHGTTEGEGQSEDHGTTEGEGKSEDHGTTEGEGQSQDHGTTEGEGQSEDHGTQEGEVK 739
            S  HGT      S  H T      S  H T      S  H T      S  H T
Sbjct: 930  SSAHGTPSTPADSSAHSTPSTPADSSAHSTPSTPADSSAHSTPSTPVDSSAHSTPSTPAD 989

Query: 740  SDEHGTTEGEGQSEDHGTTEGEGQSEDHGTTEGEVKSEDHGTTEGEGQSEDHGTQEGEVK 799
            S  H T      S  H T      S  H T    V S  H T      S  HGT
Sbjct: 990  SSAHSTPSTPADSSAHSTPSTPADSSAHSTPSTPVDSSAHSTPSTPADSSAHGTPSTPAD 1049

Query: 800  SDEHGTTEGEGQSEDHGTQEGEGKAEDHGTTEGEGQSEDHSTSEGEVRSDEHGTNDVKED 859
            S  H T      S  H T      +  HGT      S  HST      S  HGT     D
Sbjct: 1050 SSAHSTPSTPVDSSAHSTPSTPADSSAHGTPSTPADSSAHSTPSTPADSSAHGTPSTPAD 1109

Query: 860  T 860
            +
Sbjct: 1110 S 1110
```

Query: 121612134, Subject: 57237813, Rank: #5

Although the sequences are nearly identical, and they both map to models 0042501 and 0042480 from SUPERFAMILY, the alignments to those models do not overlap. For example, the query matches model positions 1:148 from 0042501, while the subject matches model positions 252:447, both with E-values of better than 10^{-17}. This problem could perhaps be avoided if HMMer had the option to return secondary high-scoring alignments of the same region to a different portion of the model.

```
 Score =  108 bits (271), Expect = 2e-23,   Method: Composition-based stats.
 Identities = 67/74 (90%), Positives = 67/74 (90%)

Query: 1  MEKSLLFHFRRIGVEFIIFSVYAVFSISWAATGSLMPLISNDLALNTQQATLITSMIVVA 60
          MEKSLLFHFRRIGVEFIIFSVYAVFSISWAATGSLMPLISNDLALNTQQATLITSMIVVA
Sbjct: 1  MEKSLLFHFRRIGVEFIIFSVYAVFSISWAATGSLMPLISNDLALNTQQATLITSMIVVA 60

Query: 61 KIFGASFTAFLVYK 74
          KIFGA    F   K
Sbjct: 61 KIFGAYLGLFFKRK 74
```

Query: 60418535, Subject: 116510983, Rank: #6

We would need to look at additional domains to find this homology. The hits to known families for this region of the query are to PF01381.13 (the full-length model of this PFam), PF01381.13.fs (the fragment model of this PFam), and model HTH_XRE from SMART. FastHMM does not find any hits for these models to the subject (although HMMer would have). The query also has a weaker hit to SUPERFAMILY model 0045230, which hits the subject, but FastBLAST does not consider this domain.

```
 Score =  103 bits (258), Expect = 5e-22,   Method: Composition-based stats.
 Identities = 54/98 (55%), Positives = 68/98 (69%)

Query: 1  MNDLFYHRLKELVEASGKSANQIERELGYPRNSLNNYKLGGEPSGTRLIGLSEYFNVSPK 60
          M ++FY RLK L   SGKS NQIERELGY RN+L NYK GG PSG RL+ L+ YF V P
Sbjct: 1  MENIFYLRLKALTHESGKSFNQIERELGYTRNALANYKNGGVPSGIRLMELANYFKVLPD 60

Query: 61 YLMGIIDEPNDSSAINLFKTLTQEEKKEMFIICQKWLF 98
          YL+G +   N  S  N F +LT ++K EM+++CQKW+
Sbjct: 61 YLIGKVPFENVESIENTFVSLTNKQKIEMYLLCQKWIL 98
```
